# A method for correcting breathing‐induced field fluctuations in T2*‐weighted spinal cord imaging using a respiratory trace

**DOI:** 10.1002/mrm.27664

**Published:** 2019-02-08

**Authors:** S. Johanna Vannesjo, Stuart Clare, Lars Kasper, Irene Tracey, Karla L. Miller

**Affiliations:** ^1^ Wellcome Centre for Integrative Neuroimaging, FMRIB, Department of Clinical Neurosciences University of Oxford Oxford UK; ^2^ Institute for Biomedical Engineering ETH Zurich and University of Zurich Zurich Switzerland; ^3^ Translational Neuromodeling Unit, Institute for Biomedical Engineering University of Zurich and ETH Zurich Zurich Switzerland

**Keywords:** 7T MRI, breathing‐induced field fluctuations, multi‐shot EPI, spinal cord imaging, T2* mapping

## Abstract

**Purpose:**

Spinal cord MRI at ultrahigh field is hampered by time‐varying magnetic fields associated with the breathing cycle, giving rise to ghosting artifacts in multi‐shot acquisitions. Here, we suggest a correction approach based on linking the signal from a respiratory bellows to field changes inside the spinal cord. The information is used to correct the data at the image reconstruction level.

**Methods:**

The correction was demonstrated in the context of multi‐shot T2*‐weighted imaging of the cervical spinal cord at 7T. A respiratory trace was acquired during a high‐resolution multi‐echo gradient‐echo sequence, used for structural imaging and quantitative T2* mapping, and a multi‐shot EPI time series, as would be suitable for fMRI. The coupling between the trace and the breathing‐induced fields was determined by a short calibration scan in each individual. Images were reconstructed with and without trace‐based correction.

**Results:**

In the multi‐echo acquisition, breathing‐induced fields caused severe ghosting in images with long TE, which led to a systematic underestimation of T2* in the spinal cord. The trace‐based correction reduced the ghosting and increased the estimated T2* values. Breathing‐related ghosting was also observed in the multi‐shot EPI images. The correction largely removed the ghosting, thereby improving the temporal signal‐to‐noise ratio of the time series.

**Conclusions:**

Trace‐based retrospective correction of breathing‐induced field variations can reduce ghosting and improve quantitative metrics in multi‐shot structural and functional T2*‐weighted imaging of the spinal cord. The method is straightforward to implement and does not rely on sequence modifications or additional hardware beyond a respiratory bellows.

## 1 INTRODUCTION

Spatial encoding in MRI relies on the assumption that the background magnetic field is homogeneous and stable over time. However, the presence of a subject in the scanner gives rise to both static field inhomogeneity and dynamic field fluctuations. Magnetic susceptibility differences between tissue and air cause local field distortions around interfaces.^1^ Because breathing changes the air–tissue distribution of the thorax and abdomen, the surrounding field distribution varies periodically with the breathing cycle.^2^ The time‐varying fields cause mislocalization of signal, which can manifest as image distortion, apparent motion, blurring, or ghosting, depending on the sequence. In neuroimaging, breathing‐related field fluctuations can be measured as far away as in the brain,^3^ but are particularly prominent in the spine because of the proximity to the lungs.^4^, ^5^ Indeed, breathing‐induced fields have been identified as one of the major challenges to overcome to achieve robust fMRI of the spinal cord.^6^

One approach to address the problem of field instability, that is well‐known from brain imaging, is to include a navigator readout in the acquisition.^7^ However, navigators take up time in the sequence and may prolong the minimum achievable TE and/or TR. They may also become unreliable in low signal‐to‐noise regimes, when robust phase extraction from the measured MR signal is difficult. One potential alternative to navigators is to base a correction on the signal from a respiratory bellows, which can track the state of the breathing cycle in real time. It has recently been shown that the respiratory trace can predict more than 90% of the breathing‐induced time variance of the field in the spinal cord during normal shallow breathing.^5^ A respiratory trace has previously been used as basis for real‐time shim updating in ultrahigh field brain imaging^8^ and has more recently been explored for real‐time shimming of the spinal cord at 3 T using a custom‐built 24‐channel shim coil.^9^, ^10^ However, real‐time shim updating demands specialized hardware, which is not available at most sites.

In this work, we investigate using the signal from a respiratory bellows to retrospectively correct for breathing‐induced phase variations in the acquired MR signal before image reconstruction. We base the correction on a model we have previously explored to transform the acquired respiratory trace to field variations inside the spine along the superior‐inferior (z) axis.^5^ The model parameters are determined in each individual subject using a short calibration scan of fast phase‐sensitive FLASH images. The method is straightforward to implement on standard MR systems and can be used for a broad range of acquisitions. Here we explore the correction in the context of T2*‐weighted imaging at 7T. Ultrahigh field accentuates the T2*‐weighted contrast and allows for higher resolution. However, T2*‐weighted acquisitions are particularly vulnerable to the effects of field fluctuations, and the fluctuations are stronger at higher background field strengths. While T2* is therefore challenging at ultrahigh field, it is also of particular interest in the spine. In structural imaging, T2*‐weighting provides excellent gray/white matter contrast in the spine^11^, ^12^, ^13^ and it is also the contrast underlying blood‐oxygen‐level‐dependent functional imaging. We therefore implement the correction for high‐resolution multi‐echo gradient‐echo acquisitions, used for structural imaging and quantitative T2* mapping, and time series of multi‐shot EPI images, intended for functional imaging. We focus on multi‐shot acquisitions, for both structural and functional imaging, as they are more robust against static B_0_ field inhomogeneity compared to single‐shot acquisitions, while being especially sensitive to dynamic field variations.

## 2 METHODS

All acquisitions were performed on a whole‐body 7T system (Magnetom, Siemens Healthineers, Erlangen, Germany). Imaging was performed with a volume‐transmit, 16‐channel receive cervical spine coil (Quality Electrodynamics, Mayfield Village, OH, USA) in 8 healthy volunteers (1 female, mean (range) age 34 (27‐40) years, weight 79 (55‐95) kg, height 1.81 (1.64‐1.93) m, body mass index 23.8 (20.4‐29.0) kg/m^2^), in compliance with local ethics guidelines. The volunteers were instructed to breathe regularly at a comfortable pace and to avoid swallowing during the scans. During all acquisitions, the signal from a respiratory bellows placed just below the thorax was recorded. A trigger signal from the sequence was simultaneously recorded to retrospectively synchronize the respiratory trace, R(t), with the acquired imaging data. No low‐pass filter was applied to the trace, but the mean offset was subtracted for each scan to remove slow baseline drifts between scans.

### 2.1 Trace‐based correction

**Figure 1:**
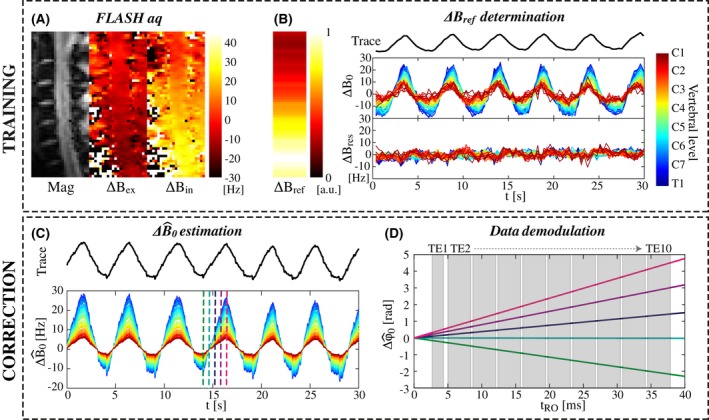
Schematic of the trace‐based correction method. A, Magnitude and phase images of a single sagittal slice are acquired with a FLASH sequence (data shown for subject 4). The phase images are here shown at a peak of expiration ($\Delta B_{\mathrm{ex}}$) and inspiration ($\Delta B_{\mathrm{in}}$). B, The coupling parameter ($\Delta B_{\mathrm{ref}}\left(z\right)$) between the respiratory trace and the field offset ($\Delta B_{0}\left(z,t\right)$) inside the spinal cord is determined for each axial slice based on the FLASH calibration data. The residual field offsets ($\Delta B_{\mathrm{res}}\left(z,t\right)$) show that the linear model explains a large part of the measured temporal field variations. C, During later scans, the field offset ($\Delta {\hat{B}}_{0}\left(z,t\right)$) can be estimated based on $\Delta B_{\mathrm{ref}}\left(z\right)$ and the respiratory trace. The color scale to indicate vertebral level is the same as in B. D, The estimated field offset yields corresponding phase evolutions ($\Delta {\hat{\phi }}_{0}\left(z,t\right)$), here shown for a multi‐echo gradient‐echo readout at 5 different time points indicated by vertical lines in the plot in C. The acquired image data are demodulated by the estimated $\Delta {\hat{\phi }}_{0}\left(z,t\right)$

The trace‐based correction pipeline is summarized in Figure 1. Calibration of the individual breathing‐induced spatial field profiles was performed as described in Vannesjo et al.^5^ In brief, a short calibration scan consisting of a time series of FLASH images^14^ (resolution 3.4 × 2.3 × 3.0 mm^3^, FOV 144 × 144 mm^2^, matrix size 43 × 64, TR 8 ms, TE 4.08 ms, bandwidth 240 Hz/pixel, FA 6˚, volume TR 344 ms) of a single sagittal slice through the center of the spinal cord was acquired during normal breathing (Figure 1A). The volume TR was minimized to yield sufficient temporal resolution to capture the breathing cycle. The respiratory period during calibration was in the range of 3.6 to 7.7 s in the different subjects, thus yielding 10 to 22 measurements within each respiratory cycle. The phase in each voxel, ϕ(r,t), was unwrapped over time and the time‐averaged phase, $\bar{\phi }\left(r\right)$, was subtracted. The resulting phase evolution was divided by the echo time to yield a measure of the field changes over time:

(1) $$\Delta B_{0}\left(r,t\right)=\frac{\phi \left(r,t\right)-\bar{\phi }\left(r\right)}{\gamma TE}.$$

A mask covering the spinal cord was manually defined on the FLASH magnitude image. The field within the mask was averaged in the transverse plane, yielding a time series of measured field offsets, $\Delta B_{0}\left(z,t\right)$, along the superior‐inferior (z) axis (Figure 1B). A linear model linking the respiratory trace *R(t)* to the estimated field offset, $\Delta {\hat{B}}_{0}\left(z,t\right)$, at any given z location was assumed:

(2) $$\Delta {\hat{B}}_{0}\left(z,t\right)=R\left(t\right)\Delta B_{\mathrm{ref}}\left(z\right).$$

A least‐squares fit based on 30 seconds of the calibration data was used to determine $\Delta B_{\mathrm{ref}}\left(z\right)$ from Equation 2 in each subject. Time points affected by swallowing were manually identified based on the associated field pattern^5^ and were excluded from the fit. The measured $\Delta B_{\mathrm{ref}}\left(z\right)$ was then used to estimate the field fluctuations from the respiratory trace acquired during subsequent scans (Figure 1C).

For the correction, a spatially homogeneous field offset within each transverse plane was assumed. The field offset was assumed to be static during the length of the readout train. In this case the MR signal from the object is modulated by a phase offset, $\Delta \phi_{0}$, given by (Figure 1D):

(3) $$\Delta \phi_{0}\left(z,t,t_{\mathrm{RO}}\right)=\gamma \Delta B_{0}\left(z,t\right)t_{\mathrm{RO}}$$

where $t_{\mathrm{RO}}$ denotes time since the last RF excitation, and t represents time over the full length of the MR sequence. The acquired imaging data, $s\left(z,t,t_{\mathrm{RO}}\right)$, were demodulated with the estimated phase offset, $\Delta {\hat{\phi }}_{0}\left(z,t,t_{\mathrm{RO}}\right)$, for each time point in the acquisition:

(4) $$\hat{s}\left(z,t,t_{\mathrm{RO}}\right)=s\left(z,t,t_{\mathrm{RO}}\right)e^{-i\Delta {\hat{\phi }}_{0}\left(z,t,t_{\mathrm{RO}}\right)}.$$

After demodulation, image reconstruction was performed with an iterative conjugate gradient optimization algorithm with SENSE unfolding.^15^ For comparison, image reconstruction was also performed without prior demodulation of the phase offset, to yield uncorrected images.

### 2.2 Image acquisitions

Imaging data for correction were acquired using two sequences: a multi‐echo 2D gradient‐echo sequence using a single‐line readout at each echo time, suitable for T2*‐weighted structural imaging and quantitative T2* mapping, and a single‐echo 2D EPI sequence using a segmented readout, suitable for high‐resolution functional imaging.

The multi‐echo 2D gradient‐echo sequence had the following imaging parameters: 24 axial slices, resolution 0.35 × 0.35 × 3 mm^3^, FOV 146 × 128 mm^2^, TR 1000 ms, 10 echoes: TE [3.51, 6.68, 10.37, 14.06, 17.75, 21.44, 25.13, 28.82, 32.51, 36.20] ms, bipolar readout, bandwidth 600 Hz/pixel for the first echo and 300 Hz/pixel for all later echoes, anterior‐posterior phase encoding, flip angle 46˚, T_acq_ 6:06 min. The increased bandwidth of the first echo served to minimize the first TE in order to achieve close to proton density contrast. The acquisitions were centered on the lower end of the C4 vertebra to fully cover the C3 to C6 vertebral levels. The magnitude images of the separate echoes (with and without correction) were combined with a root‐sum‐of‐squares (RSS) combination to form high‐resolution structural images. Quantitative T2*‐mapping was performed by a voxel‐wise fit of a monoexponential function to the individual echoes of the multi‐echo acquisition. The fit was performed in Matlab 2017a using the Trust‐Region algorithm of the “fit” function. A mask covering the spinal cord was created on the RSS images to enable quantitative analysis of the obtained T2* values inside the spinal cord with and without correction. The mask was obtained with a semiautomatic approach using the “sct_propseg” function of the Spinal Cord Toolbox^16^, ^17^ with manually selected starting points.

The multi‐shot 2D EPI sequence (24 axial slices, resolution 0.76 × 0.76 × 3 mm, FOV 128 × 128 mm, SENSE factor 2, 4 shots, TR 650 ms, volume TR 2.6 s, TE 14 ms, bandwidth 1144 Hz/pixel, echo spacing 1.06 ms, AP phase encoding, flip angle 42˚, 120 volumes, T_acq_ 5:17 min) was acquired in 6 of the volunteers (1 female, mean (range) age 34 (28‐40) years, weight 78 (55‐95) kg, height 1.82 (1.64‐1.93) m, body mass index 23.3 (20.4‐26.9) kg/m^2^). In order to minimize the achievable TE in light of very short T2* in the spine, the EPI sequences did not include phase correction lines for static ghost removal; this correction was performed using corresponding phase correction lines acquired separately in a phantom. A mask covering the spinal cord was extracted as described earlier. The temporal signal‐to‐noise ratio (tSNR) was calculated voxel‐wise and averaged inside the spinal cord mask with and without correction. The tSNR was defined as the ratio of the mean signal to the standard deviation of the signal over the time series.

## 3 RESULTS

**Figure 2:**
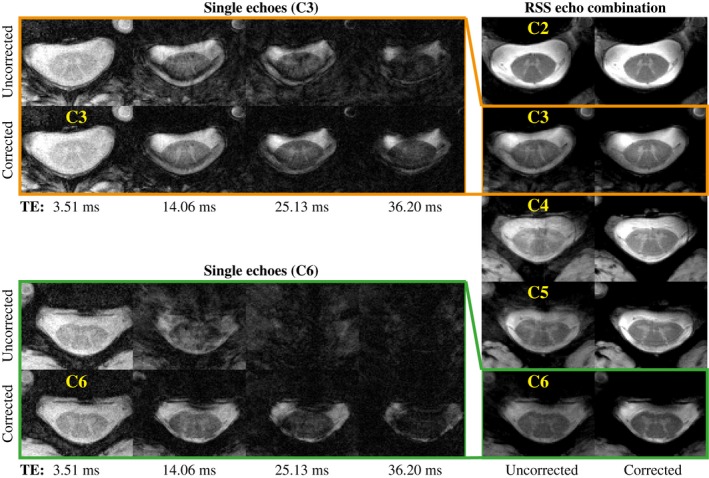
Multi‐echo gradient‐echo acquisition in one subject (subject 4). To the left are single‐echo images from the first, fourth, seventh, and tenth echoes at two different vertebral levels (C3 and C6), without and with correction. To the right are the RSS echo combination images shown at vertebral levels C2 to C6, without and with correction

Figure 2 shows single echoes and the RSS echo combination of the high‐resolution structural acquisition in one subject. In the uncorrected single‐echo images, there is an irregular ghosting pattern, which increases with echo time. The ghosting smears out the signal from the spinal cord over the image, to the degree that the depiction of the spinal cord is completely lost in later echoes in some slices. The artifacts are generally more severe toward lower levels of the cervical spine, where the field fluctuations are higher. In the RSS images, the ghosting results in a blurred appearance, reduced signal amplitude, and diminished gray/white matter contrast. With correction, the ghosting is reduced, yielding more sharply delineated structures and improved gray/white matter contrast. The dark band along the dorsal edge of the spinal cord in the C3 slice is due to local static field inhomogeneity around the intervertebral junction.

**Figure 3:**
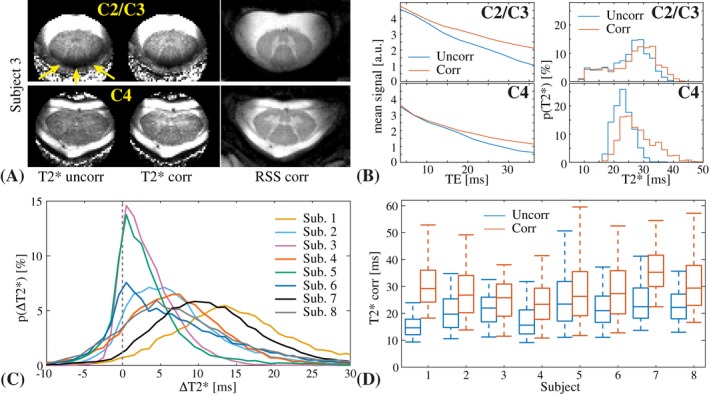
T2* quantification results from one subject (top) and a summary over all subjects (bottom). A, T2* maps without and with correction, next to the corrected RSS image, in 2 different slices from one subject. The arrows point to a band of low T2* due to static field inhomogeneity. B, Mean signal decay (left) and a histogram of the measured T2* values (right) inside the spinal cord mask without and with correction, for the 2 slices shown in A. C, Histogram of the voxel‐wise difference in the measured T2* without and with correction ($\Delta {T2}^{*}={T2}_{\mathrm{corr}}^{*}-{T2}_{\mathrm{uncorr}}^{*}$) for all subjects. D, Box plot of the T2* distribution without and with correction inside the spinal cord mask for all subjects. The center line represents the median value, the box shows the 25th and 75th percentiles, and the whiskers show the 5th and 95th percentiles

The ghosting systematically affects the T2* quantification as shown in Figure 3. The upper half of the plot (Figure 3A and B) shows the quantification results in two different slices from one subject. The mean signal within the spinal cord decays faster in the uncorrected case, resulting in a systematic underestimation of the local T2* (Figure 3B). In locations where the T2* was intrinsically low, the correction did not affect the quantification. This is evident from the upper slice, where local static field inhomogeneity due to close proximity to the C2/C3 intervertebral junction caused a marked shortening of T2* along the dorsal edge of the spinal cord (Figure 3A: yellow arrows). The T2* shortening is reflected in the lower tail of the corresponding histogram, where the uncorrected and the corrected results overlap (Figure 3B). The lower half of Figure 3 shows results for all subjects. A systematic underestimation of T2* due to field fluctuations was observed in all subjects (Figure 3C and D). The median T2* value within the full spinal cord mask was 15 to 23 ms in the uncorrected case and 23 to 35 ms with correction (Figure 3D).

**Figure 4:**
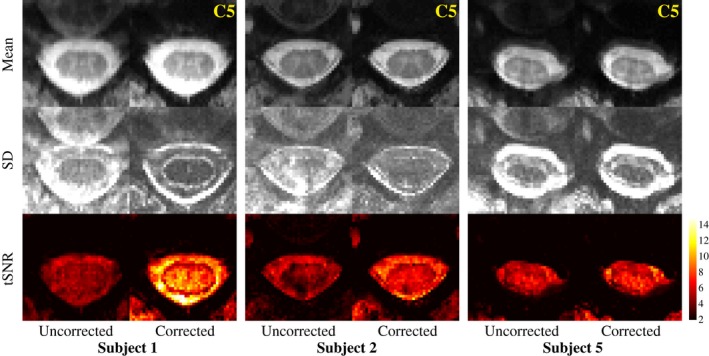
Multi‐shot EPI mean image, standard deviation (SD), and tSNR in a single axial slice at C5 midvertebral level, without and with correction in 3 different subjects

**Figure 5:**
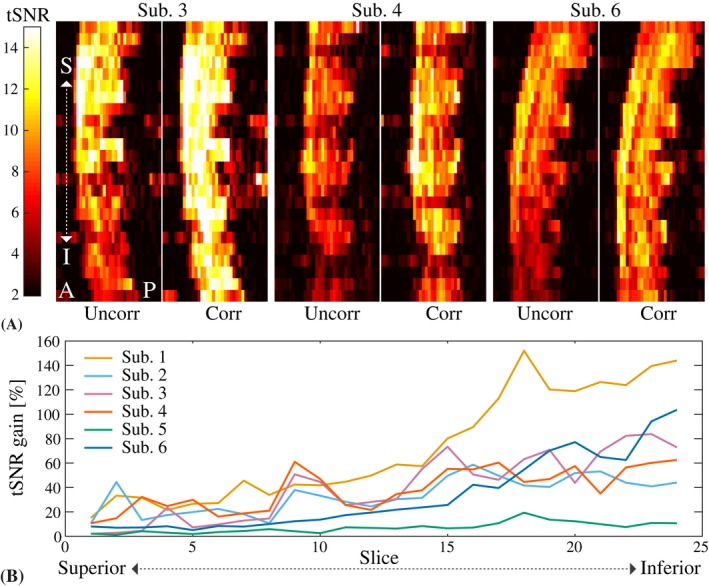
A, Sagittal tSNR maps for the multi‐shot EPI without and with correction in 3 different subjects. B, Relative tSNR gain due to the correction for all subjects. The tSNR was averaged over all voxels inside the spinal cord mask in each axial slice (slice numbers counted superior to inferior, covering approximately C2 to C6)

Figure 4 shows the multi‐shot EPI time series mean image, standard deviation, and tSNR in an axial slice at midvertebral level C5 in 3 subjects, without and with correction. The field fluctuations primarily cause data inconsistency between the different shots, leading to ghosting. The correction visibly reduced ghosting in all subjects. The magnitude of the ghosting varied considerably between subjects, as well as between volumes in the time series of a single subject. The time‐varying ghosting leads to higher standard deviation and reduced tSNR over the time series. The correction improved the tSNR inside the spinal cord in all subjects. Figure 5 shows a sagittal view of the tSNR without and with correction in 3 subjects (for completeness, we show the subjects not included in Figure 4). The improvement was larger toward lower levels of the spinal cord, where the field fluctuations are stronger. The mean tSNR within the whole spinal cord mask increased by 32% on average over the subjects (range 6%‐59%), and the tSNR at around level C6 increased by on average 69% (range 10%‐135%) (Figure 5B).

## 4 DISCUSSION

In this work, we have presented a correction for breathing‐induced field fluctuations in spinal cord T2*‐weighted imaging. Dynamic B_0_ fields are a considerable challenge in spinal cord imaging, especially at ultrahigh field.^18^ We have here demonstrated that appropriate correction is crucial for both image quality and quantitative measures in multi‐shot structural and functional acquisitions. The proposed correction utilizes a respiratory trace to remove breathing‐related phase instabilities in the acquired MR signal. The method relies on a short calibration scan, but does not require any additional hardware beyond a respiratory bellows, and is therefore straightforward to implement on standard MR systems.

The proposed method has advantages and disadvantages as compared to the more conventional approach of using a navigator readout in the sequence. Navigators measure the field directly from the tissue itself; hence they can capture field variations from various sources, such as deep or prolonged breaths, body motion, or system drift, that elude the proposed method. On the other hand, navigators require time in the sequence and therefore impose additional limits on the achievable TE and TR. Moreover, navigators depend on being able to extract a phase estimate from the MR signal reliably and are thus not useful in low‐SNR settings, such as at late echo times. Unreliable phase estimates lead to an unstable correction that may even increase artifacts. Our proposed method thus extends the set of applications and parameter settings for which it is possible to correct for breathing‐induced field fluctuations. It would be of interest to map out the relative benefit of the proposed method compared to navigators in different use cases. This is, however, beyond the scope of the current study.

Quantitative T2* mapping was one of the demonstrated applications for the proposed correction. In the uncorrected case, the T2* values in the spinal cord were systematically underestimated. This happens because the phase offset corresponding to a given field offset scales with the echo time, leading to increased ghosting in later echoes. The ghosting scatters the signal from the spinal cord over the image, thereby mimicking T2* signal loss inside the cord. The measured T2* values with correction were in good agreement with values previously reported at 7T, in a study that used a navigator echo for phase stabilization.^19^ In the context of T2* mapping, a navigator readout either replaces the first imaging echo or is appended after the last. In the former case, the early part of the decay, which carries information about the proton density of the tissue, is lost, whereas in the latter case, the navigator may have too low SNR for robust phase extraction. Both cases limit the feasible set of echo times and our navigatorless protocol is thus able to extend the observable part of the T2* decay. Measuring the full decay provides more information for the exponential fit and could potentially allow for more complicated signal models as compared to a monoexponential decay.

The second application investigated in this work was multi‐shot EPI time series for functional imaging. In brain imaging, functional acquisitions are routinely performed with single‐shot EPI. Single‐shot EPI is relatively insensitive to time‐varying field offsets, which translate into apparent motion in the time series that can be addressed retrospectively with motion correction algorithms. However, fMRI of the spinal cord at 7T has to date been conducted with 3D multi‐shot EPI acquisitions^20^, ^21^ to reduce distortion and signal dropout due to local static field inhomogeneity. Multi‐shot sequences are more susceptible to time‐varying field effects, as this causes phase inconsistencies between shots. The previous studies relied solely on postprocessing of the image time series to handle signal fluctuations of physiological origin. We here demonstrated that the tSNR of multi‐shot 2D EPI time series can be improved with correction for breathing‐induced fields, especially toward lower levels of the cervical spinal cord. This may improve the sensitivity to detect neural activation in fMRI of the spinal cord, especially in combination with postprocessing methods to further reduce the impact of signal variations of physiological origin.^22^

The proposed correction relies on a quick calibration scan to establish the relationship between respiratory trace and field variations. Here, 30 seconds of calibration data was used; however this could potentially be reduced further. In cases of atypical spinal anatomy, e.g. in scoliosis, several calibration acquisitions with shifted slice location may be required to cover the spinal cord. Also, if the subject moves considerably between scans, a recalibration may become necessary. The individual images of the calibration need to be acquired fast enough to capture the field variations of a typical breathing cycle. The parameters used here (344‐ms volume TR) are sufficient to cover normal adult respiratory rates (around 12 to 20 breaths/minute). Higher respiratory rates can, however, occur in pathological conditions, as well as physiologically in young children, in which case an even shorter volume TR may be necessary.

The proposed correction method was able to reduce ghosting artifacts greatly, but did not completely eliminate them. Residual artifacts after correction are also frequently observed with phase navigators. A number of potential reasons for incomplete correction can be identified. First, the presented model assumes a reproducible and linear relationship between respiratory trace signal and the field state. This is a good approximation during regular shallow breathing, but is less reliable during deep or irregular breathing.^5^ Second, the spatial field profile of the time‐varying fields may not be perfectly homogeneous within the transversal slice. Previous characterizations of breathing‐induced fields in the cervical spine have demonstrated a field gradient in the anterior‐posterior direction in slices through the center of the neck, and a highly nonlinear field component in slices closer to the thorax.^5^ Third, the correction only accounts for breathing‐induced fields, and not for actual motion of the subject nor for field variations from other sources. Slight actual motion of the neck associated with the breathing cycle is expected. Furthermore, swallowing induces both local motion of tissue and field variations of up to about 40 Hz.^5^ Residual ghosting due to swallowing was occasionally observed in the multi‐shot EPI time series. Further signal fluctuations may appear due to pulsatile flow of cerebrospinal fluid surrounding the cord, as well as pulsatile motion of the spinal cord itself, primarily associated with the cardiac cycle.^23^, ^24^

The trace‐based correction could potentially be expanded to account for some of the additional perturbations mentioned. For example, slice‐dependent anterior‐posterior field gradients could be estimated from the sagittal FLASH images, and included in the reconstruction by shifting the k‐space sampling points accordingly. A nonlinear model linking the trace to the field variations may be able to capture a larger range of fluctuations and breathing modes. Furthermore, additional external devices, such as NMR field probes^25^ or motion‐tracking optical devices,^26^ could yield more information about both the field state and the motion, which could then be incorporated into the reconstruction model.^27^ The correction could also be extended to further acquisition types, such as spin echo acquisitions and 3D imaging. In the former case, the phase offset is refocused at TE, but there is still a linearly varying phase over the readout that may impact long readouts, such as EPI. In 3D imaging, the correction needs to represent the breathing‐induced field variations within the full imaging volume. As a first approximation, the center frequency offset within the slab can be used. A more accurate model can include linear gradients in the foot‐head and anterior‐posterior directions.

A further improvement to the current implementation of the correction method would be to eliminate the manual steps in the processing of the field calibration data, i.e. the spinal cord mask creation and the exclusion of time points affected by swallowing. This would be a crucial step to allow for integration into standard acquisition protocols. Potentially this could be achieved with the Spinal Cord Toolbox17 for masking, combined with automatic outlier detection in the field time courses.
